# Microcystin-LR regulates circadian clock and antioxidant gene expression in cultured rat cardiomyocytes

**DOI:** 10.1186/s11658-018-0115-z

**Published:** 2018-10-10

**Authors:** Yonghua Xu, Xiangmin Wang, Surong Jiang, Chen Men, Di Xu, Yan Guo, Jun Wu

**Affiliations:** 1 Department of General Surgery, The Fourth Affiliated Hospital of Nantong University, Yancheng, 224006 China; 2 Department of Geriatric Cardiology, The First Affiliated Hospital of Nanjing Medical University, 300 Guangzhou Rd, Nanjing, 210029 China

**Keywords:** Circadian clock, Antioxidant defense, Microcystin-LR, Heart

## Abstract

**Background:**

Microcystins are waterborne environmental toxins that induce oxidative stress and cause injuries in the heart. On the other hand, many physiological processes, including antioxidant defense, are under precise control by the mammalian circadian clock.

**Results:**

In the present study, we evaluated the effect of microcystin-LR (MC-LR) on the rhythmic expression patterns of circadian and antioxidant genes in rat cardiomyocytes using the serum shock technique. We found that a non-toxic dose (10 μm) of MC-LR decreased the amplitudes of rhythmic patterns of clock genes, while it increased the expression levels of antioxidant genes.

**Conclusions:**

Our results indicate an influence of MC-LR on the circadian clock system and clock-controlled antioxidant genes, which will shed some light on the explanation of heart toxicity induced by MC-LR from the viewpoint of chronobiology.

## Introduction

A great number of physiological events in living organisms exhibit 24-h circadian rhythm fluctuations [1–3]. These intrinsic biological rhythms are driven by a circadian clock and are dominantly governed by light-dark (LD) and feeding cycles. The mammalian circadian clocks comprise a central clock and slave oscillators. The central clock is located in the hypothalamic suprachiasmatic nucleus (SCN), while slave oscillators are found in various tissues, which can be driven by the central clock via behavioral and neuroendocrine signals [4]. At the molecular level, autoregulatory transcriptional translational feedback loops involving several clock genes build up intracellular clocks. Nearly 43% of all genes are expressed in a circadian manner in the whole genome [5].

Microcystin (MC) is a kind of cyclic heptapeptide compound, which is the most ubiquitously distributed hepatotoxin. It is mainly produced by freshwater species of *Microcystis*. MCs were reported to cause illness or even death in animals and humans [6]. Mechanistically, MCs inhibit both protein serine/threonine phosphatases-1 and 2A [7, 8], enhance formation of reactive oxygen species (ROS) and interact with mitochondrial ATP synthase, aldehyde dehydrogenase and also with mitochondrial oxidative phosphorylation [9, 10]. Microcystin-LR (MC-LR) is the most common and well-known toxic variant among the microcystin family [11]. Hepatocytes become apoptotic and necrotic when exposed to large doses of MC-LR. On the other hand, MC-LR contributes to the development of liver cancer and damages the heart under chronic poisoning.

When oxidative stress is induced by MCs, intracellular antioxidant defense is evoked, trying to remove its harmful consequences. Several key enzymes such as catalase, superoxide dismutase, and heme oxygenase play a vital role in this process. It is interesting that the levels of these antioxidant enzymes and low molecular weight antioxidants such as glutathione also show circadian rhythms, which leads to time dependence of the sensitivity to environmental oxidants [12, 13]. Hence, we hypothesized that MC-LR may alter the rhythmic expression patterns of clock and antioxidant genes in the heart, which contributes to its myocardial toxic effects.

## Materials and methods

### Ethical statement

This article does not describe any studies with human participants or animals performed by any of the authors.

### Cell culture

The H9C2 cardiomyocytes were grown in Dulbecco’s modified essential medium (DMEM; high glucose; Gibco-Invitrogen, Carlsbad, USA), supplemented with 10% (*v*/v) fetal bovine serum (FBS; Gibco-Invitrogen), 25 mmol/L HEPES (pH 7.4), penicillin (100 U/mL), and streptomycin (100 mg/mL) at 37 °C in a humidified atmosphere of 95% air and 5% CO_2_.

### MTT assay

The effect of MC-LR (Taiwan Algal Science, Taiwan) on H9C2 cell viability was analyzed by using an MTT assay. Cells were seeded in 96-well plates (1 × 10^4^ cells per well). MC-LR (0.1–100 μm) or vehicle (0.0001–0.1% DMSO) was added to cells and incubated for 24 h. After that, MTT (0.2 mg/ml) was added to each well and incubated for 4 h. The supernatant was removed and the formazan crystals were dissolved in DMSO. Cell viability was assessed by measuring the absorbance at 550 nm using a microplate reader.

### Serum shock

To assess the rhythmic gene expression in H9C2 cells, serum shock experiments were performed (Balsalobre et al. 1998). In brief, medium was replaced with DMEM plus 50% horse serum (*t* = 0). After 2 h, H9C2 cells were washed once with PBS buffer and incubated with serum-free DMEM with or without 10 μM MC-LR. Cells were collected at the indicated time-points and subjected to reverse transcription-quantitative PCR (RT-qPCR) analysis.

### RT-qPCR

Total RNA from H9C2 cells was extracted using Trizol reagent (Invitrogen, Carlsbad, CA). 1 μg of total RNA was reverse-transcribed into complementary DNA. A primer for rat 18 s rRNA was included for normalization. mRNA levels were quantified by real-time RT-PCR using SYBR premix Ex Taq (Takara, Japan). Samples were amplified using the Mastercycler ep realplex2 system (Eppendorf, Hamburg, Germany). Primer sequences are available upon request. The fold change value was calculated by applying the following equation: fold change = 2^-(ΔΔCt)^.

### Statistical analysis

Groups of data are presented as mean ± standard error. Data were analyzed using two-way ANOVA followed by Fisher’s LSD post-hoc test. Calculations were performed using the statistical package SPSS for Windows version 12.5S (SPSS, Chicago, USA). A value of *P* < 0.05 was considered statistically significant.

## Results

**Fig. 1:**
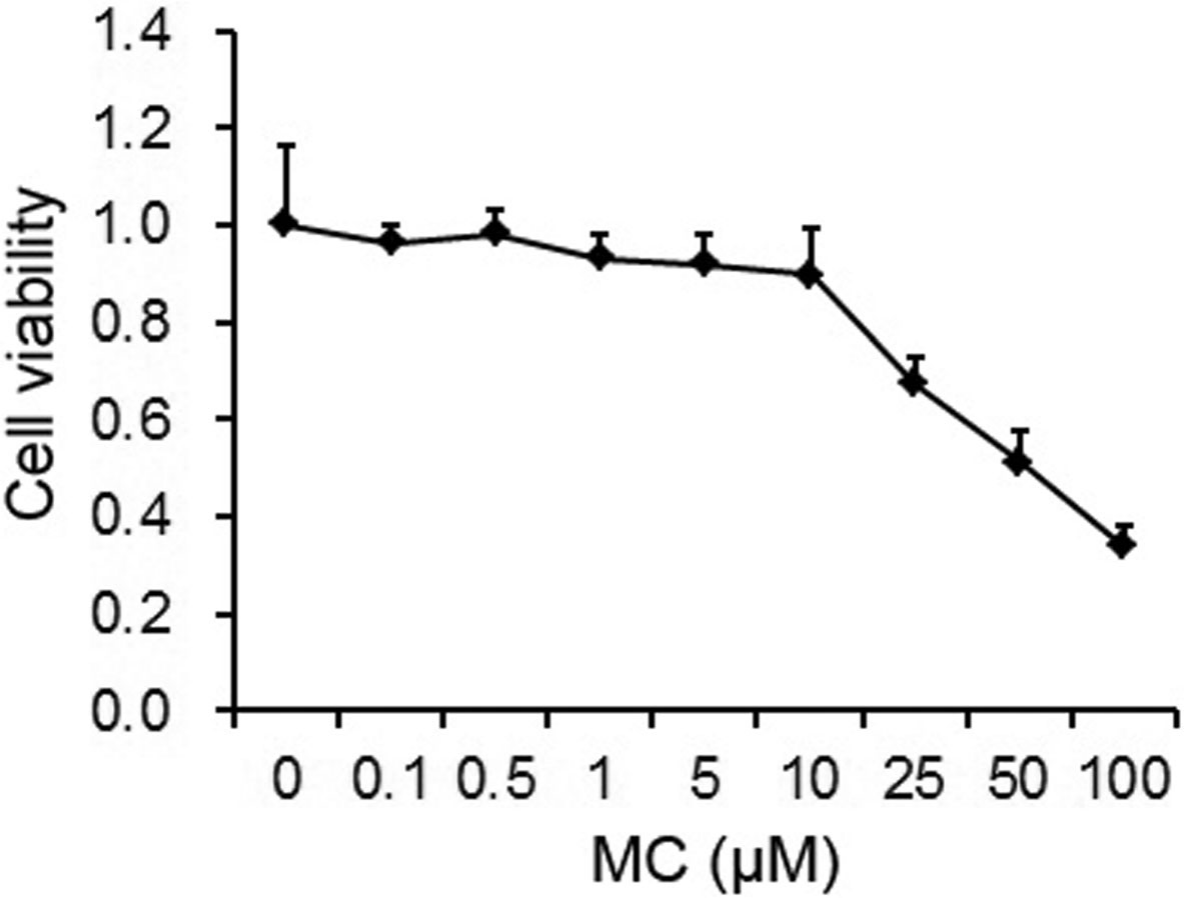
Effects of MC-LR on H9C2 cell viability. H9C2 cells seeded in 96-well plates were treated with increasing concentrations of MC-LR for 24 h before the MTT assay

We first investigated the toxicity of MC-LR towards rat H9C2 cardiomyocytes by measuring cell viability using the MTT test. As shown in Fig. 1, MC-LR was cytotoxic when the concentration was higher than 10 μm. Therefore, a 10 μm dose of MC-LR was chosen for the following experiments because it was the highest dose that did not change cell viability significantly.

**Fig. 2:**
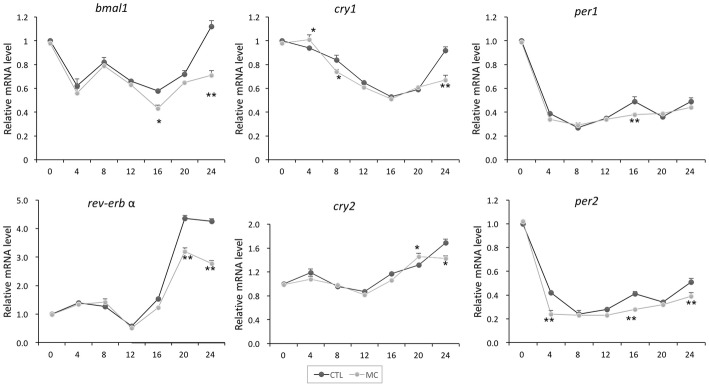
Temporal expression of circadian clock genes after MC-LR addition in H9C2 cells by serum shock. H9C2 cells were exposed to DMEM plus 50% horse serum for 2 h. At time zero, MC-LR (10 μM) was added to the medium and the cells were cultured until collection at the times of 0 h, 4 h, 8 h, 12 h, 16 h, 20 h, and 24 h. The relative mRNA levels were determined by RT-qPCR analysis. The signals obtained for each mRNA were normalized to those of 18sRNA. Data are mean ± SEM of 3 independent experiments. ^*^P < 0.05 and ^**^P < 0.01 versus control (vehicle-treated)

Next, we induced rhythmic expression of circadian clock and antioxidant genes in H9C2 cardiomyocytes by serum shock. To determine whether MC-LR would affect the expression of the clock and antioxidant genes, a 10 μm dose of MC-LR was added to the medium and the cells were cultured until collection at the indicated times. As shown in Fig. 2, a significant diurnal oscillation for all the clock genes examined was observed (repeated measures ANOVA analysis, *P* < 0.05), except for *bmal1* and *per1*. *bmal1* showed semidiurnal rather than diurnal rhythm, which may be due to the difference between the in vitro cell culture condition and the in vivo physiological condition. The *per1* gene did not display an obvious circadian oscillation, which is in line with previous findings [14]. Of note, all the genes shared a similar rhythmic expression pattern. Their mRNA levels were decreased in the first several hours, and then gradually increased. MC-LR treatment did not alter the phase of oscillation patterns of clock genes, but inhibited the amplitudes at most checked time-points. Such inhibitions became robust when the treatment lasted.

**Fig. 3:**
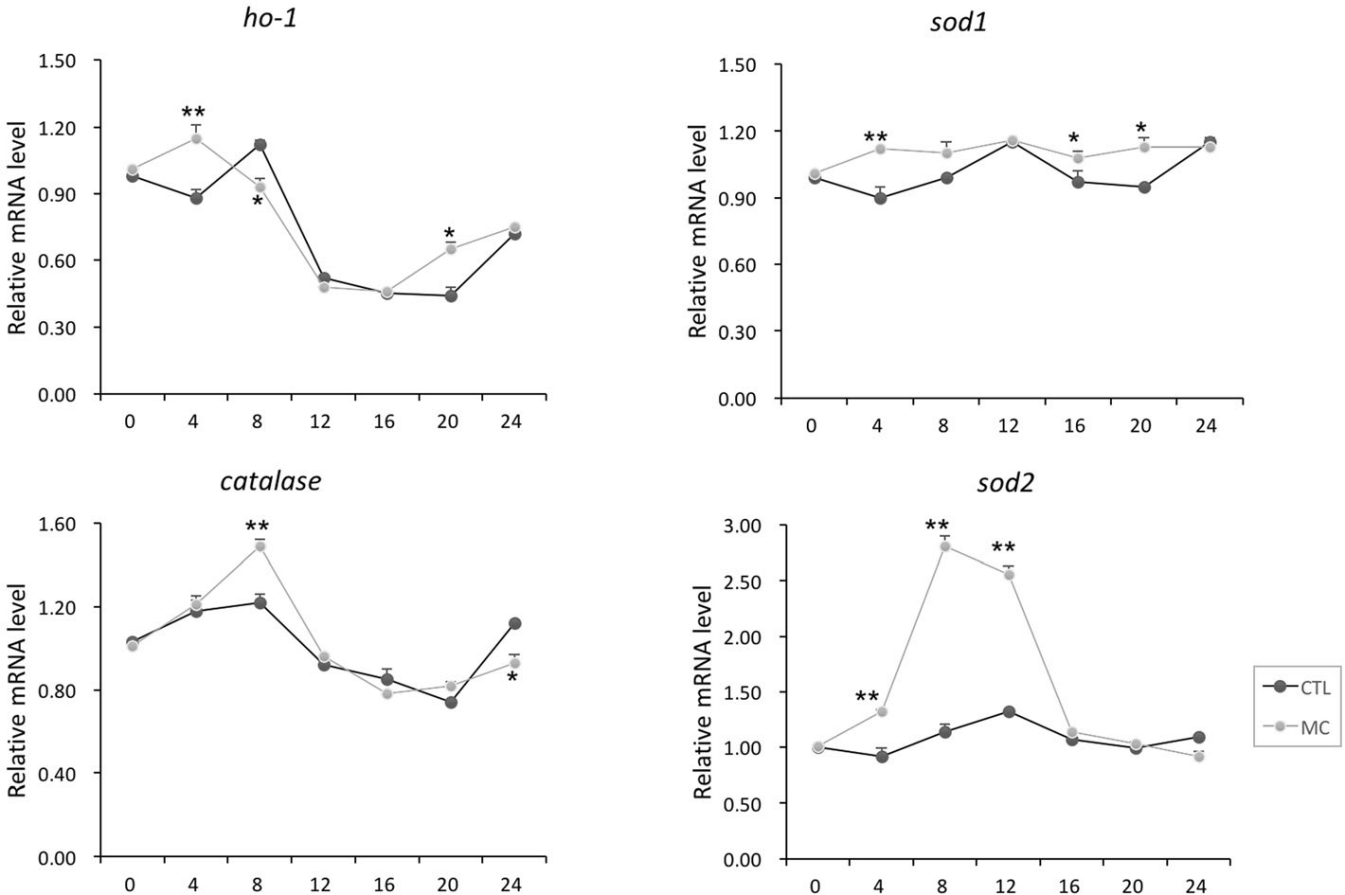
Temporal expression of antioxidant genes after MC-LR addition in H9C2 cells by serum shock. The samples and analysis were the same as described in Fig. 2. Data are mean ± SEM of 3 independent experiments. ^*^P < 0.05 and ^**^P < 0.01 versus control (vehicle-treated)

**Fig. 4:**
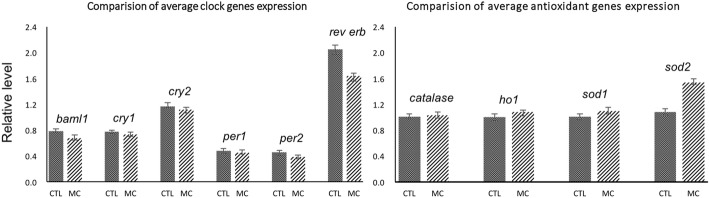
Average 0–24 h rhythmic expression levels of clock and clock-controlled antioxidant genes after MC-LR addition in H9C2 cells by serum shock. Data are mean ± SEM of 3 independent experiments

Since MC-LR can increase ROS formation, it is interesting to examine the changes of rhythmic expression of antioxidant genes when MC-LR treatment was present. As shown in Fig. 3, the mRNA expression levels of heme oxygenase-1 (*ho-1*) and *catalase* showed robust diurnal oscillations, while the expression of superoxide dismutase 1 (*sod1*) and *sod2* remained constant during a 24 h period. On the other hand, MC-LR treatment significantly increased the expression levels of all examined antioxidant genes. Of note, the induction of *sod2* by MC-LR reached the maximum levels at 8 h and 12 h. However, MC-LR only moderately increased, or did not change, *sod2* expression at other examined time-points. Such time-dependent regulation seemed to make *sod2* oscillate. Moreover, we analyzed the average 0–24 h rhythmic expression levels of clock and clock-controlled antioxidant genes separately via average bar graphs. As shown in Fig. 4, we found that MC-LR treatment slightly suppressed the clock genes, while it increased the expression of antioxidant genes.

## Discussion

In this study, we found for the first time that MC-LR regulates clock and antioxidant genes in rat H9C2 cardiomyocytes. The amplitude of rhythmic expression of clock genes was decreased by MC-LR. Similar phenotypes have been observed in various pathophysiological settings, such as aging, obesity, and cancer [15–17], implicating the impairments of inner microenvironment homeostasis within cells. Our findings support this notion and provide evidence that MC-LR causes cellular toxicity by suppressing the robustness of the circadian clock.

The transcriptional control of genes involved in antioxidant defense and the oxidative stress response by the circadian clock can occur in several ways. Some antioxidant genes, such as *GPx-1*, *Cat*, *Sod-1*, and *TXNRD-1*, can be directly regulated by the circadian clock transcriptional factors BMAL1/CLOCK, RORs, and Rev-Erbs. They can also be regulated by clock-controlled transcriptional factors. Finally, regulation may occur through clock-dependent control of epigenetic modification by clock-controlled chromatin-modifying enzymes. [18, 19] As to the antioxidant genes, the expression levels of *sod1* and *sod2* were stable and did not oscillate. The constitutive expression of *sod1* and *sod2* in various tissues has been reported [20] and demonstrates the importance of these two enzymes in the cellular antioxidant defense. They are the main quenchers for superoxide, which is the most active and harmful species. On the other hand, all the examined antioxidant genes showed increased expression levels upon MC-LR treatment. We believe that this phenotype reflected the heavier burden of oxidative stress within cells, and therefore higher amounts of antioxidant enzymes were required to relieve more severe oxidative stress.

Although it is known that MC-LR impairs the heart function and alters the structure of heart muscle [21], the mechanism involved remains unclear. Recent studies have found that the homeostasis of the circadian clock and the function of the cardiovascular system are tightly integrated [22]. The results of our study indicate an influence of MC-LR on the circadian clock system and clock-controlled antioxidant genes, which will shed some light on the explanation of heart toxicity induced by MC-LR from the viewpoint of chronobiology.

## Conclusions

Collectively, our results indicated that in addition to the previously reported toxicological effects, MC-LR also has a profound influence on modulation of the circadian clock system of cardiomyocytes. Therefore, the systemic toxicity of MC-LR may show a diurnal oscillation and should be re-evaluated from the viewpoint of chronobiology.

## Abbreviations

bmal1: Brain and muscle Arnt-like protein-1
cry1: Cryptochromes 1
DMEM: Dulbecco’s modified Eagle’s medium
DMSO: Dimethyl sulfoxide
FBS: Fetal bovine serum
GPx-1: Glutathione peroxidase 1
ho-1: Heme oxygenase-1
LD: Light-dark
MC: Microcystin
MTT: 3-(4,5-dimethylthiazol-2-yl)-2,5-diphenyltetrazolium bromide
PBS: Phosphate buffered saline
per1: Period 1
RORs: Retinoic acid receptor-related orphan receptors
ROS: Reactive oxygen species
RT-qPCR: Reverse transcription-quantitative polymerase chain reaction
SCN: Suprachiasmatic nucleus
sod-1: Superoxide dismutase 1
TXNRD-1: Thioredoxin reductase 1
